# Case report: Poor prognosis or poor prognostication?

**DOI:** 10.1017/S1478951524001512

**Published:** 2024-10-31

**Authors:** Jacqueline Tschanz, Rida Khan, Eduardo Bruera

**Affiliations:** Department of Palliative Care, Rehabilitation, and Integrative Medicine, The University of Texas MD Anderson Cancer Center, Houston, TX, US

**Keywords:** Prognostication, prognosis, palliative care, advanced cancer, end of life

## Abstract

**Objectives:**

This case highlights the limitations of current prognostication and communication in clinical practice.

**Methods:**

We report a case of a 50 year old patient with metastatic melanoma following admission to intensive care unit and later transferred to palliative care unit for end-of-life care.

**Results:**

The patient had clinical improvement despite signs of predictors of death and was later transferred back to care of oncology team.

**Significance of results:**

Physicians frequently overestimate or underestimate survival time which can be distressing to patients and families. There is need for further research to improve the accuracy of these tools for the sake of our patients and their families.

## Introduction

Although prognostication for survival can be challenging, this information can be critical to helping patients and families make plans. Despite how difficult this survival prognostication can be, research has shown that patients and their families want as much information as clinicians are able to provide. Most patients with advanced cancer want to know their prognosis including survival time(Enzinger et al. 2015; Hagerty et al. 2004; Umezawa et al. 2015). Caregivers of patients at end of life have been shown to want more information about the process communicated to them (Sercekus 2014).

Clinician prediction of survival is the most common approach and is generally approached in 3 ways: (1) temporal approach (how long will the patient live?) (2) the surprise question (would I be surprised if the patient died by [time]?), and probabilistic approach (what is the probability the patient will live until [time]?) (Perez-Cruz et al. 2014). There are various scoring tools physicians have used to try to assist with end-of-life prognostication such as Palliative Performance Scale (PPS), Palliative Prognostic Index, Palliative Prognostic Score, Glasgow Prognostic Score have been used most frequently. Unfortunately, these systems are not always accurate and are not routinely used in clinical practice.

Death within 3 days can be more accurately diagnosed, generally assessed with physical exam findings including pulselessness, mandibular breathing, hyperextension of neck, death rattle, Cheyne-stokes breathing, and drooping of the nasolabial folds (Hui et al. 2015; Mori et al. 2022).

Systematic reviews have shown physicians tend to overestimate survival (Cheon et al. 2016). Providing incorrect prognosis for patients can be distressing for patients and caregivers. In the following case, the patient was given a predicted survival but had an unexpected outcome.

## Case

A man in his 50’s with metastatic melanoma which has progressed through multiple lines of therapy and found to have metastatic disease to brain presented to a tertiary care center for a second opinion. The patient arrived by car at the cancer center for an outpatient visit, but he had a seizure en-route. He became unconscious with clonic movements of his limbs. He was then seen in the emergency department of the cancer center. Despite the use of a benzodiazepine, steroids, and levetiracetam, the patient remained unresponsive and was transferred to the intensive care unit. Brain imaging showed metastatic lesions causing a midline shift and entrapment of a portion of the temporal lobe. After further treatment did not lead to recovery, the patient’s wife was told he would not be a candidate for further therapy and transfer to the palliative care unit (PCU) for comfort care was recommended.

Prior to transfer to the PCU, the patient was on dexamethasone 4 mg every 6 hours. In the PCU the patient had flattening of his nasolabial folds and continued to be unresponsive. Patient was continuing to have seizures requiring lorazepam, however his wife wanted less use of the sedating medication and medical team thus tried more aggressive steroid dose of 12 mg of dexamethasone twice daily. A family meeting was held with physician and multidisciplinary team of a counselor and a chaplain to help support her and communicate the patient’s imminent death based on the flattening of his nasolabial folds and PPS of 10.

Initial week in the PCU was uneventful requiring little symptom manage as patient was unresponsive. However, the patient progressively regained consciousness in the week that followed. After 2 weeks in the PCU, he had the energy to sit up and was eating solid food. The melanoma team was reconsulted to assess for treatment candidacy. Repeat magnetic resonance imaging showed decrease in swelling around tumors and resolution of prior midline shift. The patient was transferred out of the PCU and treated under the care of oncology teams for several months.

## Discussion

This case highlights that prognostication, even when backed by scientific studies, is not always accurate. A prior study has shown that doctors overestimate the time a patient has remaining (Cheon et al. 2016), but this case showcases an example of experienced palliative care specialists underestimating the prognosis. The uncertainty regarding survival can be distressing to family members who are trying their best to process the condition of a loved one while simultaneously making the necessary arrangements (Hui et al. 2019).

In our case, the patient was determined to be imminently dying based on the low PPS and flattening of nasolabial folds. These findings have been shown to be indicative of death in less than 3 days in 94% of cases (Hui et al. 2015). Prognostication in cancer can be particularly difficult. Patients who die of diseases related to organ failure such as CHF, COPD, or CKD have markers of the function of the organ that can assist in prognosis and show decline. Cancer patients tend to die of complications, most frequently, sepsis, thrombosis, or arrhythmias without warnings of decline (Bruera et al. 2015). The nature and onset of those complications cannot be predicted by tumor mass. Another major reason for difficulty in prognosis is the continuous improvement in cancer therapies (Larkin et al. 2019). Prognosis for malignant melanoma has changed greatly in the last decade from medial survival of 11 months in 2013 to 24 months in 2021 (van Not et al. 2024). This blurring of therapeutic limits is occurring for multiple hematological and solid tumors (Knight et al. 2023). When our team reconsulted the melanoma oncologists they concluded that the patient might benefit from further treatment. One of the advantages of palliative care teams in acute care facilities is the seamless interaction with specialists that make consultation much less difficult for the patient and family as compared to hospice revocation.

Our patient later clinically improved and revoked comfort care while in the PCU, electing to pursue more treatment offered by the melanoma team. One study showed that around 18% of patients who are discharged from hospice do so to resume disease directed therapy (Russell et al. 2017). Discharge from hospice can be a difficult experience for patients and caregivers (Wu and Volker 2019). Though our patient was able to pursue disease directed therapy after electing comfort care, one study showed that patients who choose to revoke hospice to pursue treatment have higher mortality rates in the next 6 months following revocation (LeSage et al. 2014). Another study showed that aggressive medical treatment at end of life was associated with worse caregiver bereavement (Wright et al. 2008). Improving our existing survival prognostic models might prevent this kind of distress for patients and families.

Physicians who care for patients toward the end of life are experienced in keeping families updated day to day, as clinical status can change quickly and without notice. Current prognostic models can help guide discussion with family members, but physicians need to be aware of the risk of the anchoring bias, in which the medical team only makes decisions based on the initial presented information, rather than reevaluating the situation completely even when things do not go as expected (Rehana and Huda 2021). Continuously reassessing day to day and keeping families updated is of vital importance. A bias toward palliative care is less common (Erel et al. 2022), but as this case highlights, is still possible.

A number of studies have shown the need for better communication for families from the medical team at this delicate time. Some of the issues family members had include not receiving an explanation for how long the patient and family could talk and having a feeling of uncertainty caused by vague explanations about future changes (Mori et al. 2022). Though the inaccuracies can be distressing, forgoing these survival prognostic models completely is also not the answer. Not giving family members a chance to prepare for the patient’s death could result in a sense of having unfinished business, which leads to higher depression and grief scores (Mori et al. 2022).

Uncertainty in prognosis cannot be helped, however there are actions the medical team can take to mitigate the effects of uncertainty for family members. For instance, it is common practice at our institution to use large margins in our temporal and percentage estimations due to this uncertainty (Cheon et al. 2016), including phrases like “hours to days” and “days to weeks.” Once patients have elected for comfort care, we do not pursue invasive measures, like labs or imaging, which could potentially help diagnose any complications that come up. Instead, we assure the patient and family members that we will manage any distress that is the consequence of complication with symptom management. Another important strategy is to remind caregivers that because we are imperfect at prognostication (Chen et al. 2023), any plans or decisions need to be made immediately. Giving the caregivers this opportunity allows them to care for themselves by prioritizing their rest and nutrition so they can care for the patient better as well.

## Conclusion

Survival prognostication is valuable to families. Despite the vast number of survival prognostication models available, physicians still overestimate or underestimate survival. Further research should focus on specific prognostic factors and their rate of accuracy so the existing survival prognostic models can be improved.
